# Supplementary material for: Probing the activity of cysteine cathepsins in inflammatory bowel diseases
Source: Sci Rep. 2025 Dec 23;16:2739. doi: 10.1038/s41598-025-32489-7 (PMC12824247; doi:10.1038/s41598-025-32489-7)

Figure 1A (L117)

Citrate PBS

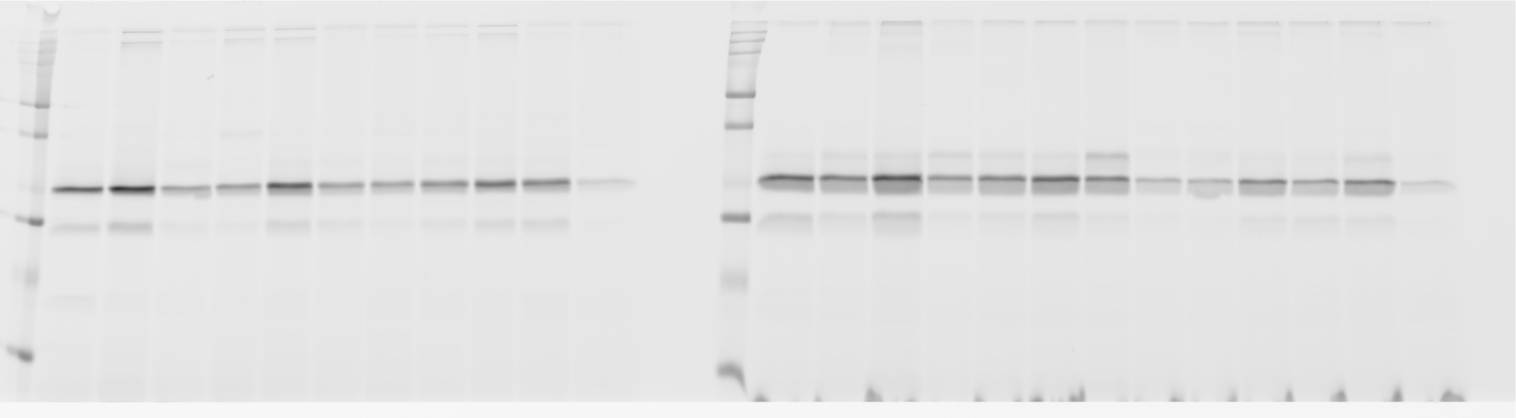

Figure 1C (Z12)
BMV157 (top)

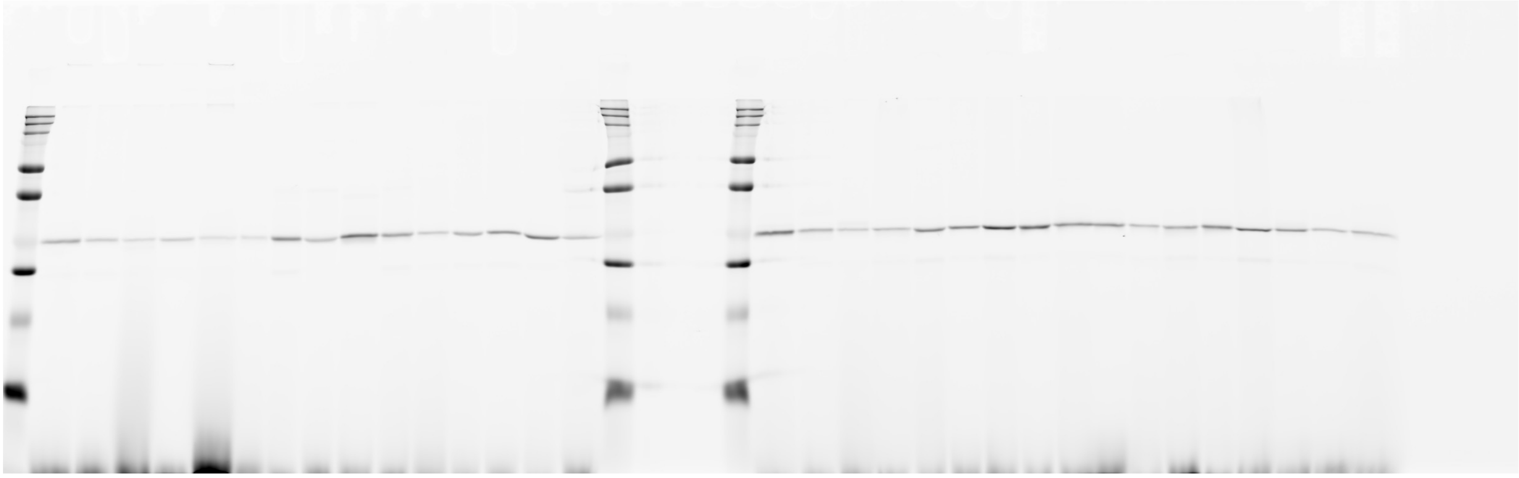

Cat S immunoblot (bottom)

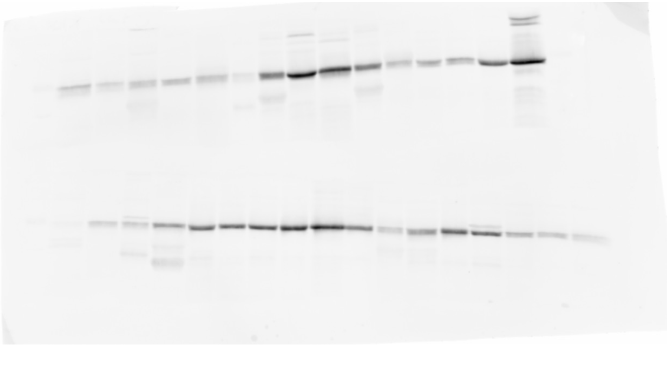

All samples run on one gel, scanned for Cy5 (top), then cut and transferred onto to one membrane for cat S blot (bottom)

Figure 1F
L183

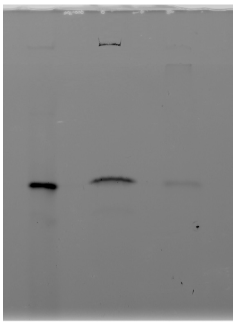

Figure 3A (N169)

Figure 3B

Figure 3C

Figure 3H(N186)

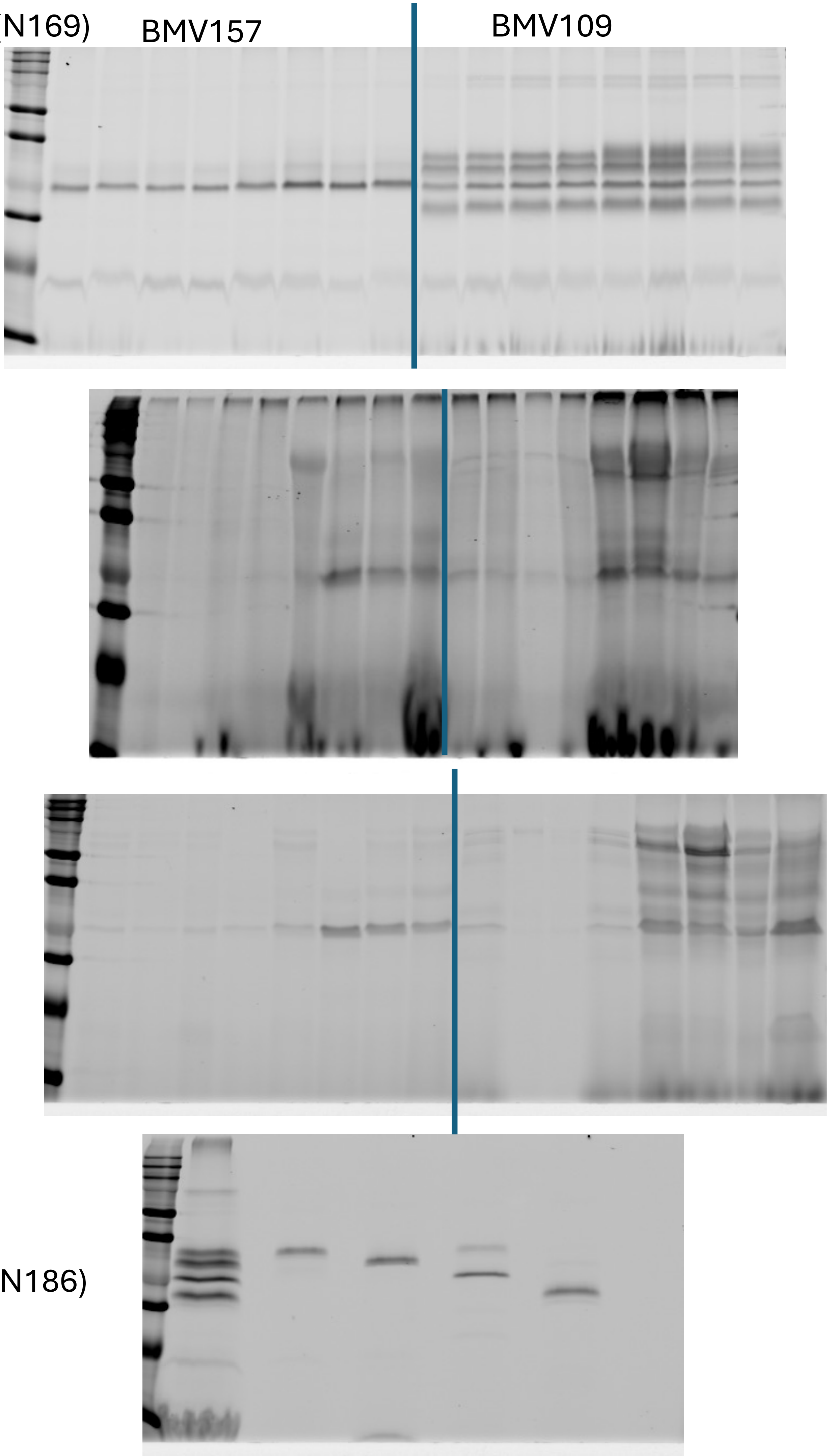

Figure 4A (N173)

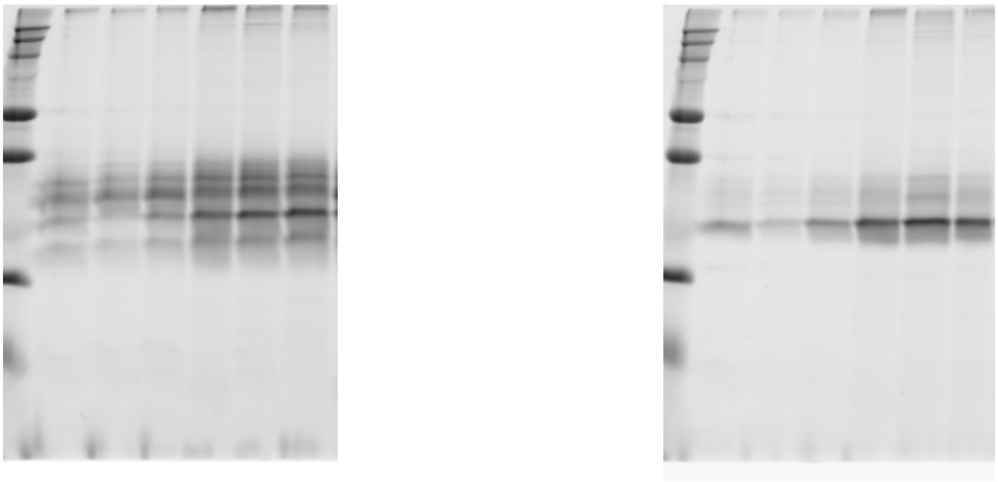

Figure 4B

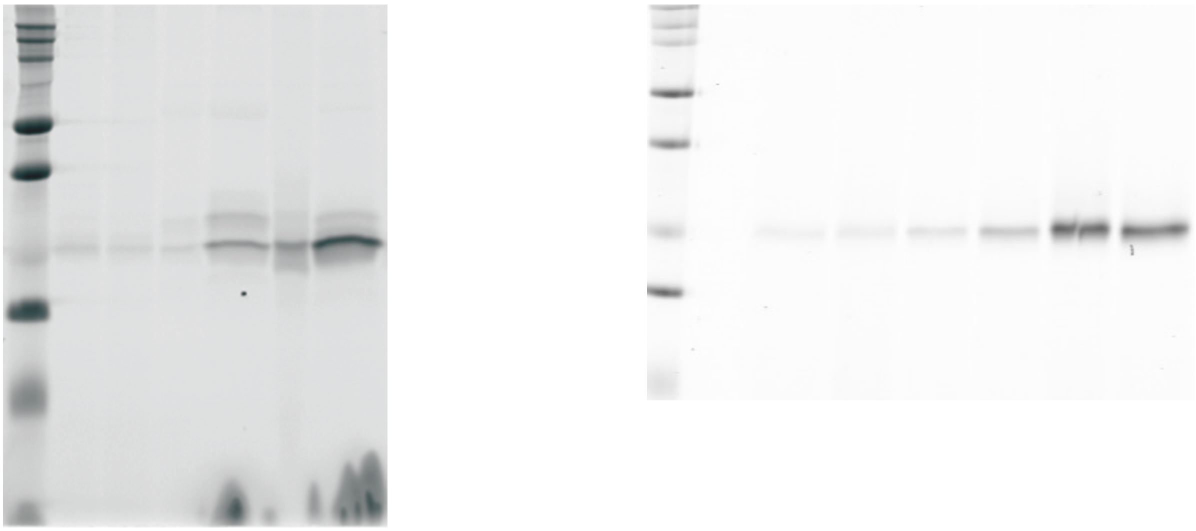

Figure 4C

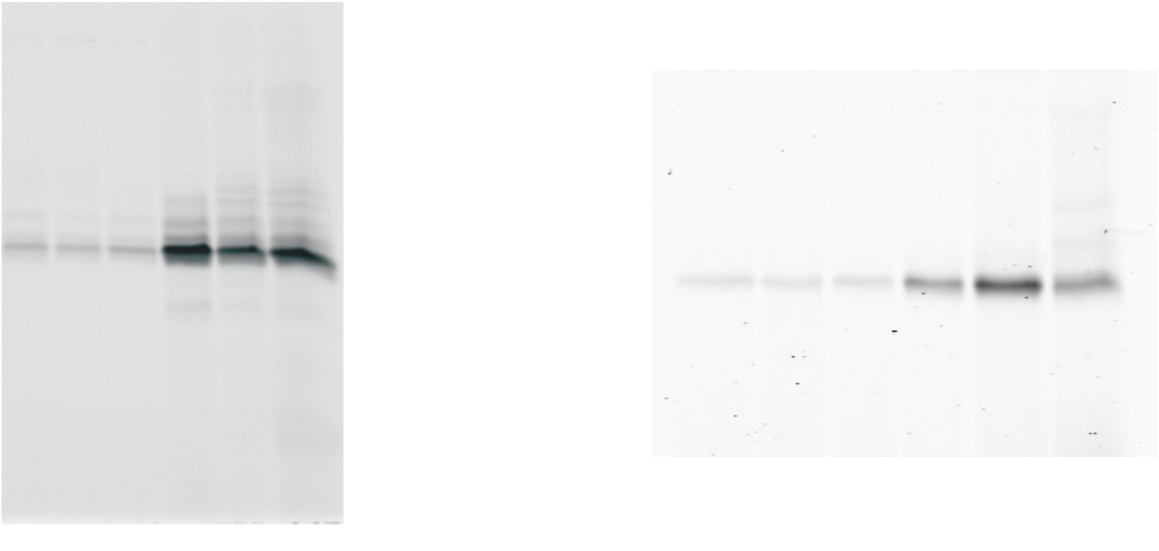

Figure 4H

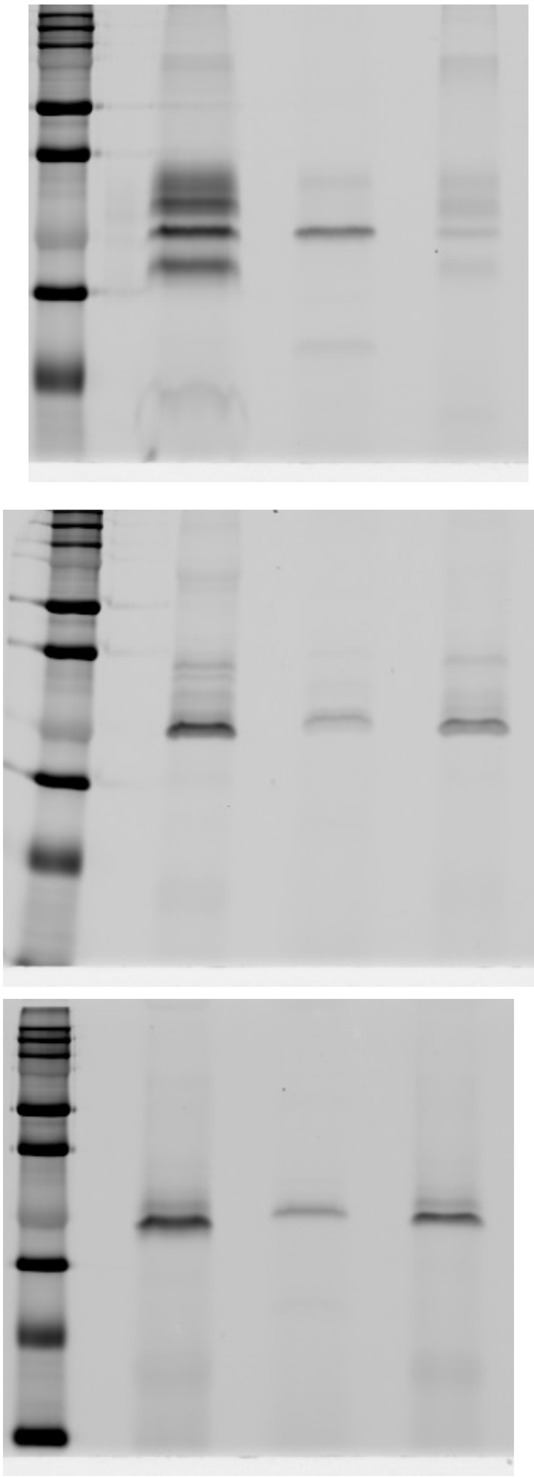

Figure 4I (N41)

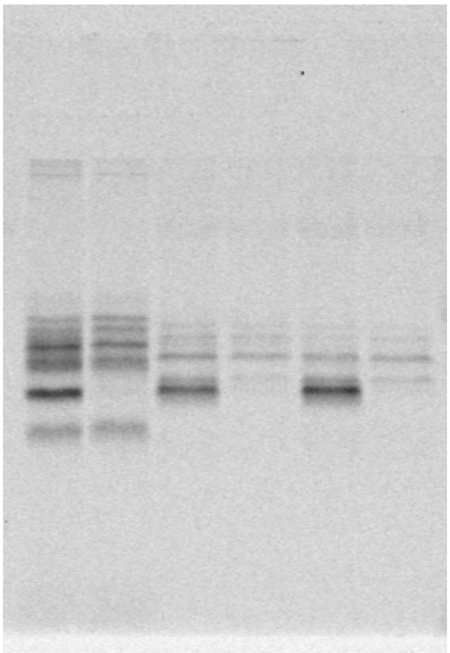

Figure 4J

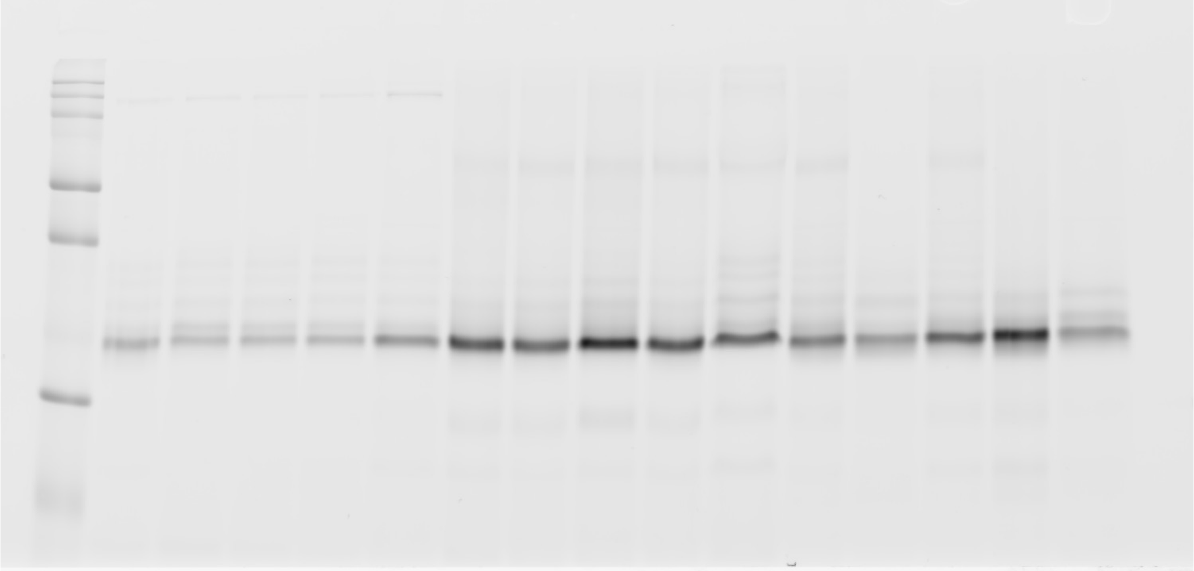

Figure 4L (N116)

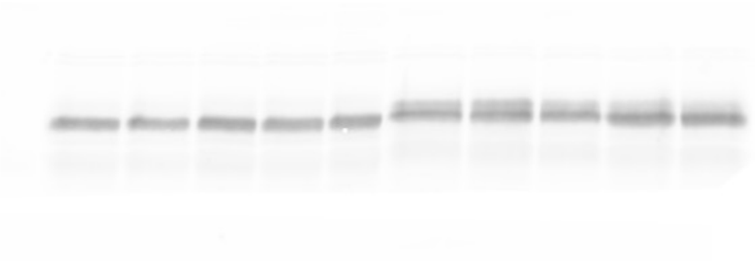

Figure 5A (N186)

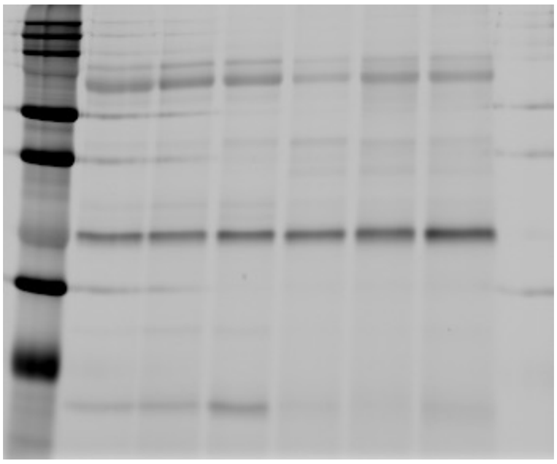

Figure 5B (N186)

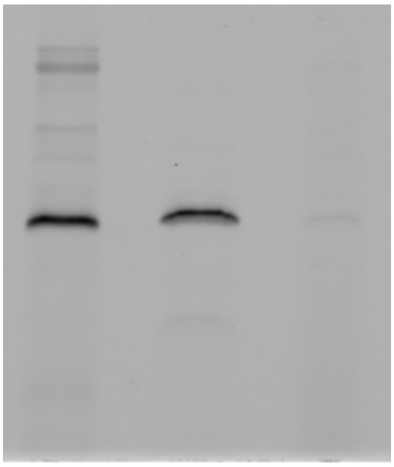

Figure 5C (L40)

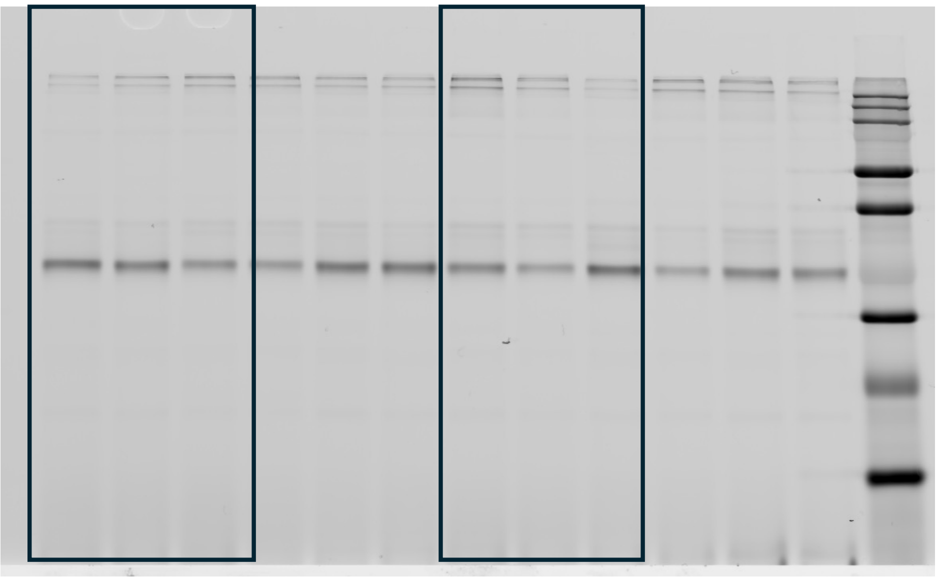

Figure 5D (L20)

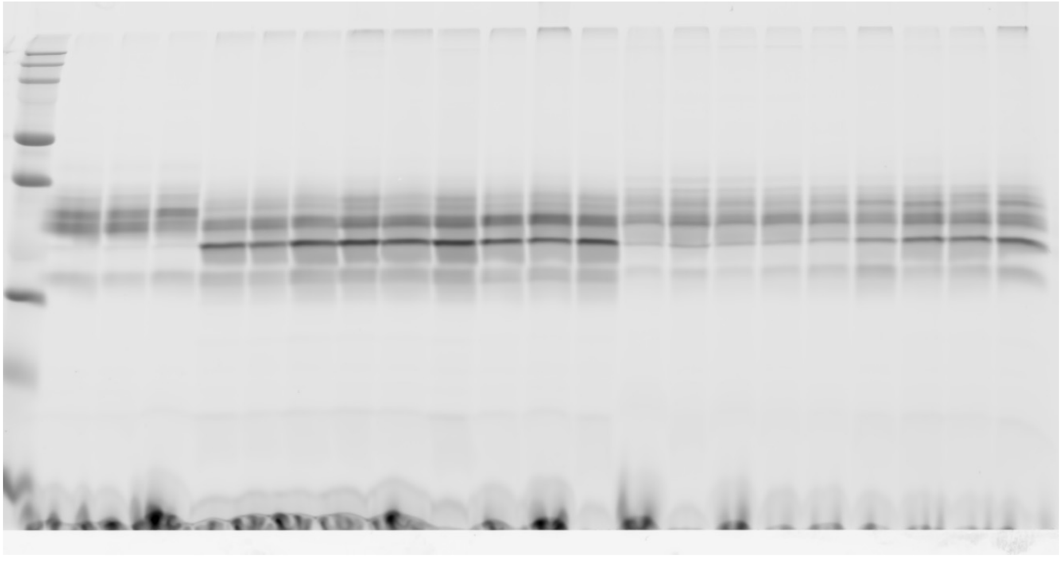

Figure 6A (L4)

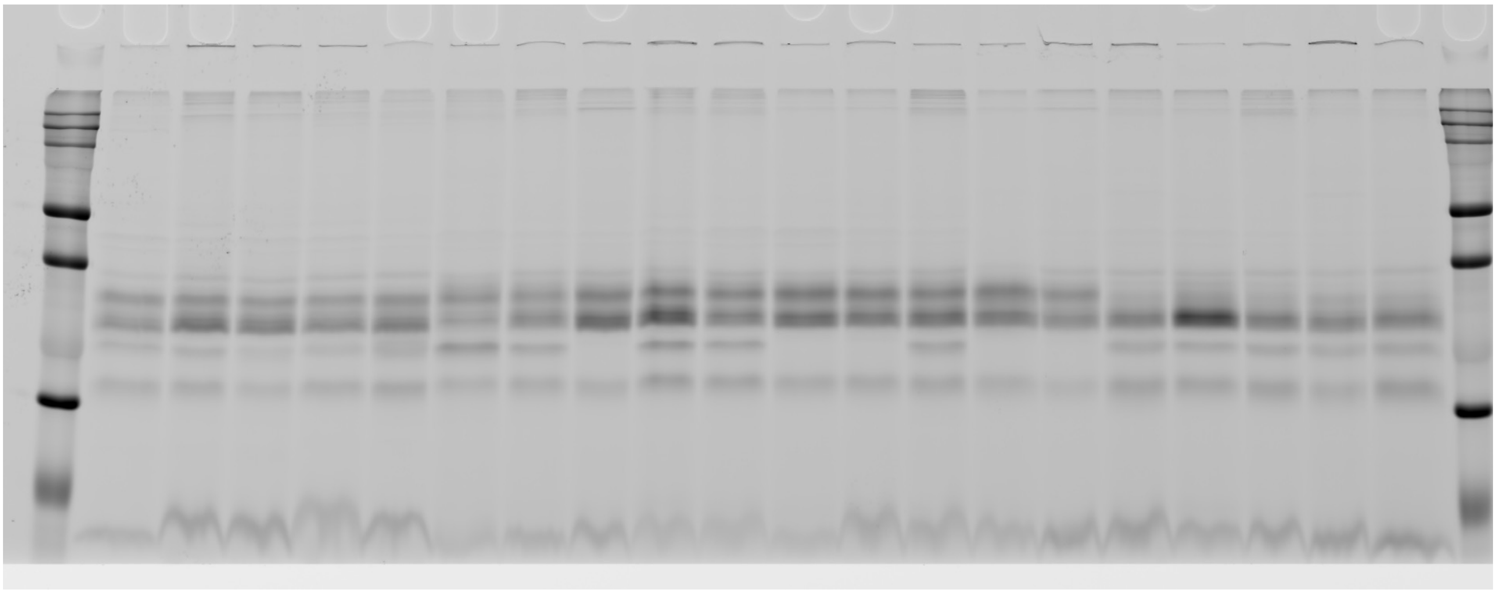

Figure 6B

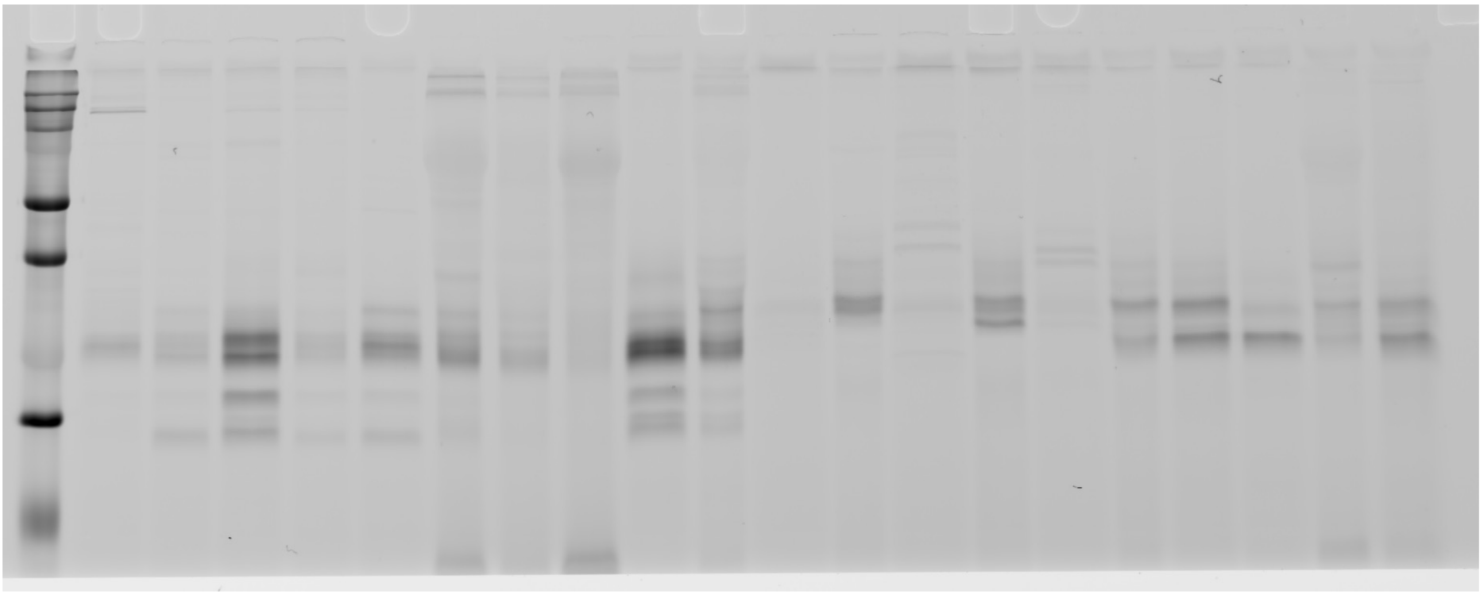

Figure 6C

Day 1

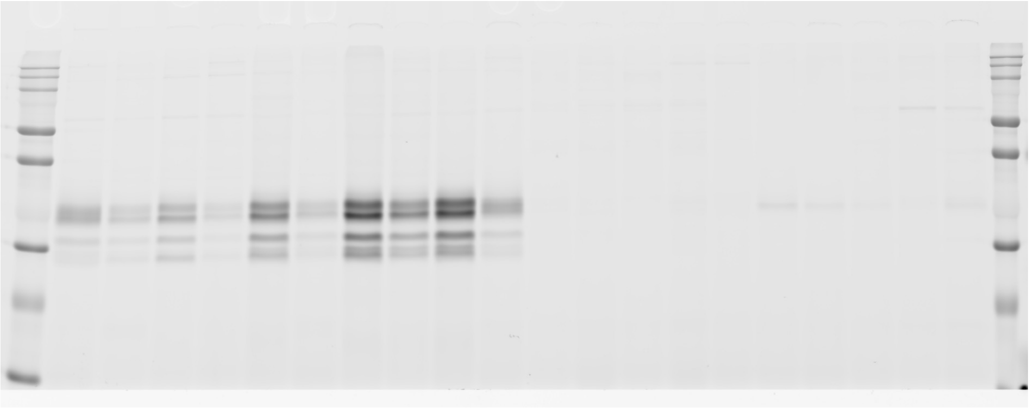

Day 3

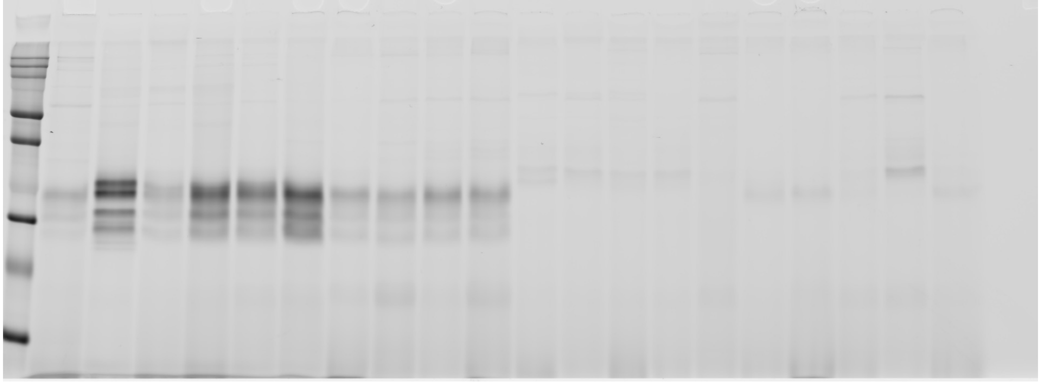

Day 4

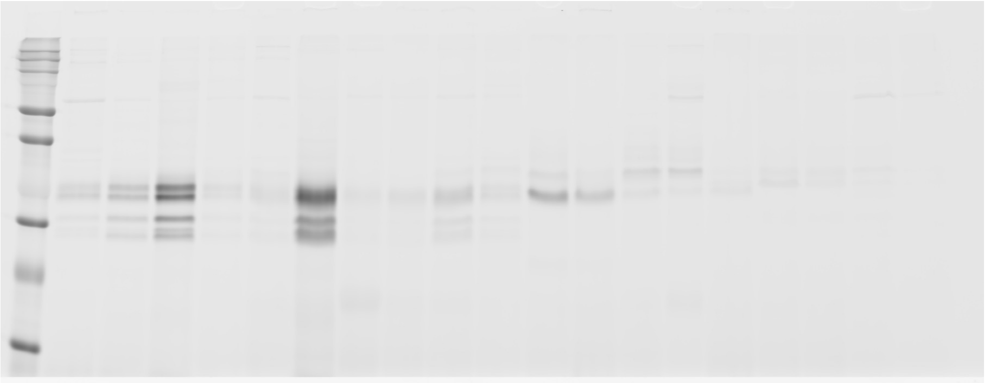

Day5

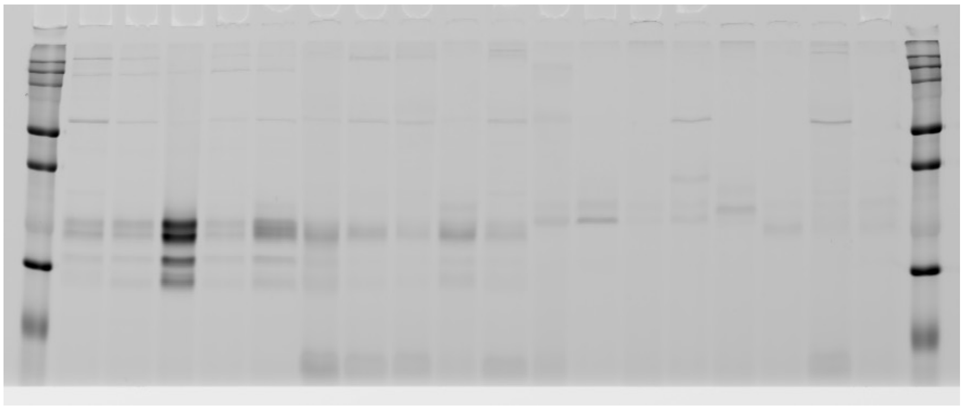

Figure 7H

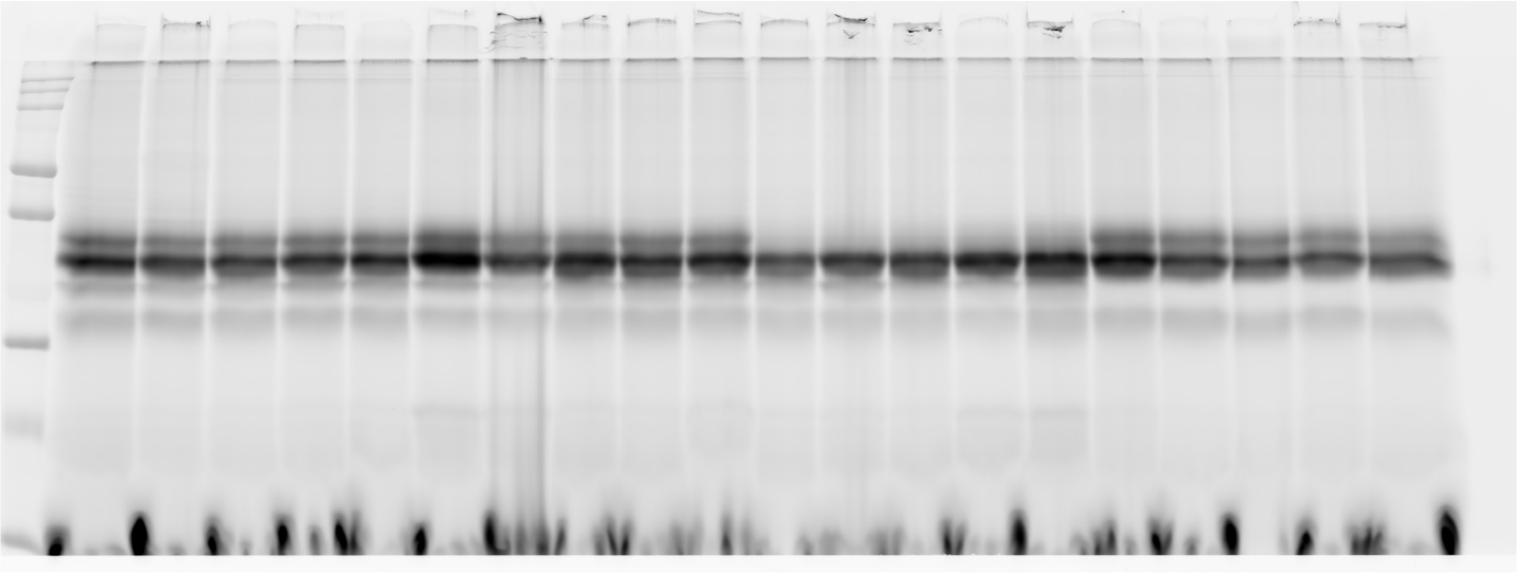

Figure 9A (N204)

Figure 9B

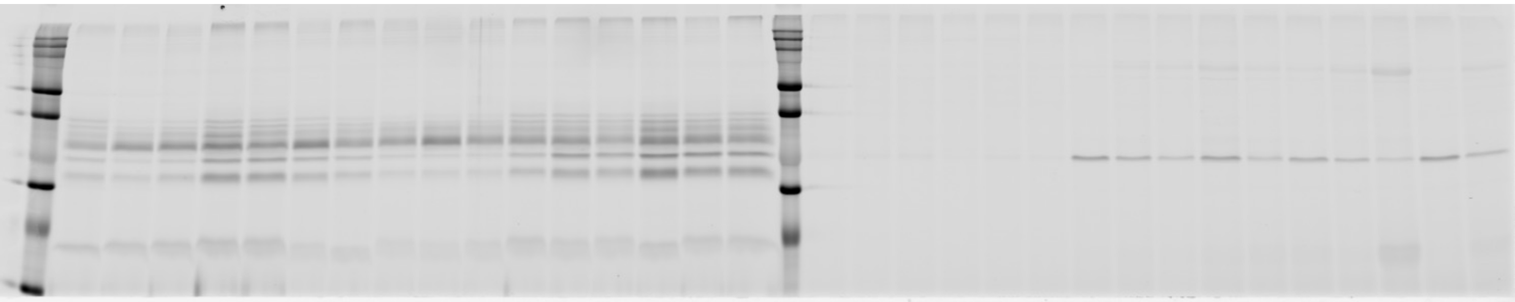

Figure 9F

Cat S

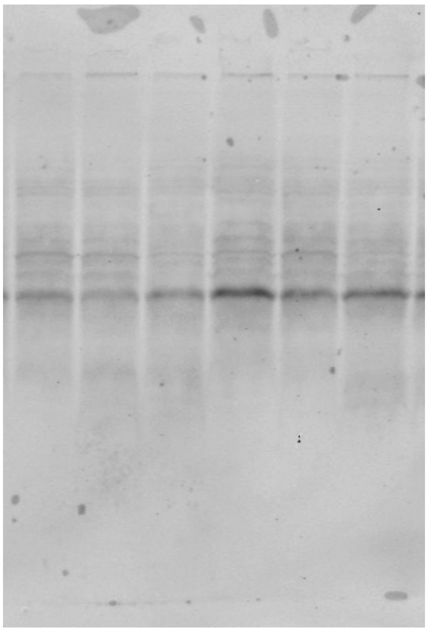

Cat L

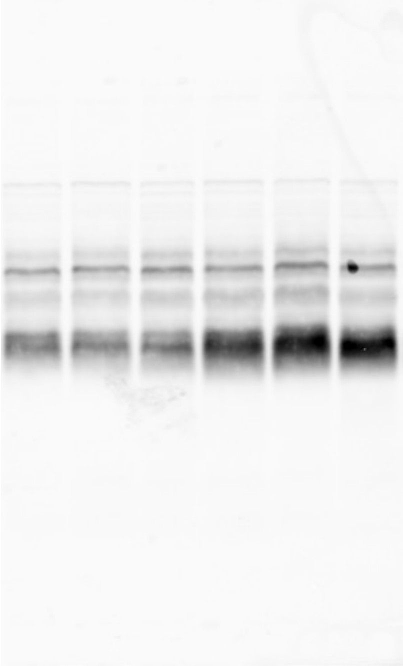

Figure 9H (N186)

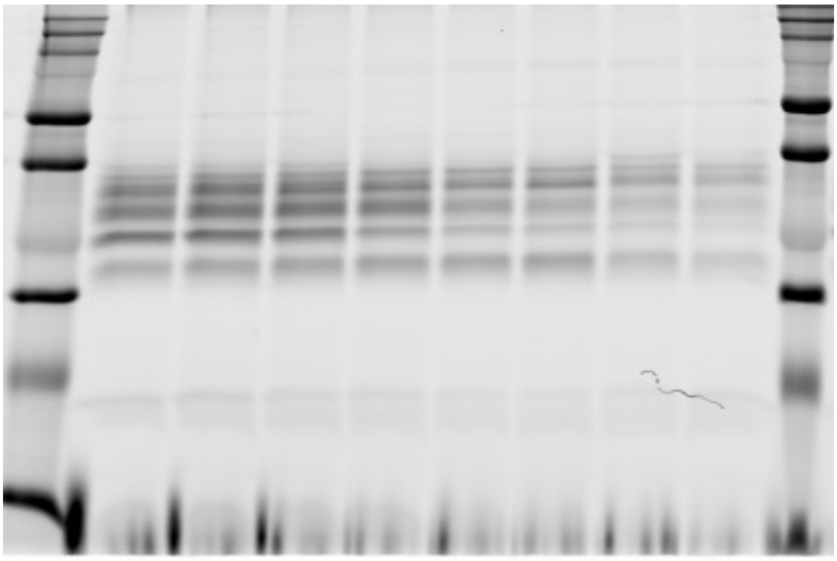

Supplement: Supplementary file 1 — Supplementary Information. [file 41598_2025_32489_MOESM1_ESM.pdf]
